# Rapid Breathing as a Pain Obtunder in Surgery, Etc., Etc.

**Published:** 1881-07

**Authors:** W. G. A. Bonwill


### ARTICLE II.

### *Rapid Breathing as a Pain Obtunder in Minor Surgery, Obstetrics, the General Practice of Medicine, and of Dentistry.*

BY W. G. A. BONWILL, D. D. S.

Read before the Philadelphia County Medical Society, May, 1880.

Through the kind invitation of your Directors, I am present to give you the history of "rapid breathing" as an analgesic agent, as well as my experience therein since I first discovered it. It is with no little feeling of modesty that I appear before such a learned and honorable body of physicians and surgeons, and I accept the privilege as a high compliment. I trust the same liberal spirit which prompted you to call this subject to the light of investigation will not forsake you when you have heard all I have to say and you sit in judgment thereon. Sufficient time has now elapsed since the first promulgation of the subject for the shafts of ridicule to be well nigh spent (which is the common logic used to crush out all new ideas,) and it is to be expected that gentlemen will look upon it with all the charity of a learned body, and not be too hasty to condemn what they have had but little chance to investigate; and, of course, have not practiced with that success which can only come from an intelligent understanding of its application and *modus operandi*.

Knowing the history of past discoveries, I was well prepared for the crucible. I could not hope to be an exception. But, so far, the medical profession have extended me more favor than I have received at the hands of the dental profession.

My first conception of the analgesic property of a pain obtunder in contradistinction to its anæsthetic effect which finally led to the discovery of the inhalation of common air by " rapid breathing," was in 1855, while performing upon my own teeth certain operations which gave me intense pain, and I could not afford to hurt myself, without a resort to ether and chloroform. These agents had been known so short a time that no one was specially familiar with their action. Without knowing whether I could take chloroform administered by myself, and at the same time perform with skill the excavation of extremely sensitive dentine or tooth-bone, as if no anæsthetic had been taken, and not be conscious of pain, was more than the experience of medical men at the time could assure me. But, having a love for investigation of the unknown, I prepared myself for the ordeal. By degrees I took the chloroform until I began to feel very plainly its primary effects, and knowing that I must soon be unconscious, I applied the excavator to the carious tooth, and, to my surprise, found no pain whatever, but the sense of touch and hearing were marvelously intensified. The small cavity seemed as large as a half bushel; the excavator more the size of an ax; and the sound was equally magnified. That I might not be mistaken, I repeated the operation until I was confident that anæsthetics possessed a power not hitherto known, that of analgesia. To be doubly certain, I gave it in my practice, in many cases with the same happy results, which saved me from the risks incident to the secondary effects of anæsthetics, and which answered for all the purposes of extracting from one to four teeth. Not satisfied with any advance longer than I could find a better plan, I experimented with the galvanic current (to and fro) by so applying the poles that I substituted a stronger impression by electricity from the nerve centers or ganglia to the periphery, than was made from the periphery to the brain. This was so much of a success that I threw aside chloroform and ether, and removed the living nerve of a tooth with instruments instead of using arsenic, and for excavating sensitive caries in the teeth, preparatory to filling, as well as many teeth extracted by it. But this was short-lived, for it led to another step. Sometimes I would inflict severe pain in cases of congested pulps or its hasty application, or pushing it to do too much, when my patient invariably would draw or inhale the breath *very forcibly and rapidly.* I was struck with the repeated coincident, and was led to exclaim: "Nature's anæsthetic." This then reminded me of boyhood's bruises. The involuntary action of every one who has a finger hurt is to place it to the mouth and draw violently in the air and hold it for an instant, and again repeat it until the pain is subdued. The same action of the lungs occurs, except more powerfully, in young children who take to crying when hurt. It will be noticed they breathe very rapidly while furiously crying, which soon allays the irritation, and sleep comes as a sequel. Witness also when one is suddenly startled, how violently the breath is taken, which gives relief. The same thing occurs in the lower animals when pain is being inflicted at the hand of man.

This was advance No. 3, and so sure was I of this new discovery, that I at once made an application while removing decay from an extremely sensitive tooth. To be successful, I found I must make the patient take the start, and I would follow with a thrust from the excavator, which move would be accomplished before the lungs could be inflated. This was repeated for at least a minute, until the operation was completed, I always following immediately or synchronously with the inhalation.

This led to step No. 4, which resulted in its application to the extracting of teeth and other operations in minor surgery.

Up to this time I had believed the sole effect of the rapid inhalation was due to mere diversion of the will, and this was the only way nature could so violently exert herself—that of controlling the involuntary action of the lungs to her uses by the *safety valve*, under the voluntary movement.

The constant breathing of the patient for thirty seconds to a minute left him in a condition of body and mind resembling the effects of ether and chloroform in their primary stages. I could but argue that the prolonged breathing each time had done it: and, if so, then there must be some specific effect over and above the mere division by the will. To what could it be due? To the air alone, which went in excess into the lungs in the course of a minute! Why did I not then immediately grasp the idea of its broader application as now claimed for it? It was too much, gentlemen, for that hour. Enough had been done in this fourth step of conception to rest in the womb of time, until by evolution a higher step could be made at the maturity of the child. Being self-satisfied with my own baby, I watched and caressed it until it could take care of itself, and my mind was again free for another conception.

The births at first seemed to come at short intervals: but see how long it was between the fourth and the fifth birth. It was soon after that my mind became involved in inventions—a hereditary outgrowth—and the electric mallet and then the dental engine, the parent of your surgical engine, to be found in the principal hospitals of this city, took such posseession of my whole soul, that my air analgesic was left slumbering. It was not until August, 1875—nineteen years after—that it again came up in full force, without any previous warning.

This time it was no law of association that revived it; but it seemed the whispering of some one in the air—some ethereal spirit, if you please—which instituted it and advanced the following problem: "Nitrous oxide gas is composed of the same element as ordinary air, with a larger equivalent of oxygen, except it is a chemical compound, not a mechanical mixture, and its anæsthetic effects are said to be due to the excess of oxygen. If this be a fact, then why can you not produce a similar effect by rapid breathing for a minute, more or less, by which a larger quantity of oxygen is presented in the lungs for absorption by the blood?"

This query was soon answered by asking myself another. "If the rapid inhalation of air into the lungs does not increase the heart's action and cause it to drive the blood in exact ratio to the inhalations, then *I can* produce partial anæsthesia from this excess of oxygen brought about by the voluntary movements over the ordinary involuntary action of the lungs." The next question was: "Will my heart be affected by this excess of air in the lungs to such an extent that there will be a full reciprocity between them?" Without making any trial of it, I argued that, while there is no other muscular movement than that of the chest as under the control of the will, and as nature has given to the will the perfect control over the lungs to supply more or less air as is demanded by the pneumogastric nerve for the immediate wants of the economy, when the *involuntary action* is not sufficient; and the heart not being under the control of the will, and its action never accelerated or diminished except by a specific poison, or from the general activity of the person in violent running or working, the blood is forced into the heart faster and it must get rid of it, when a larger supply of oxygen is demanded and rapid breathing must occur, or asphyxia result. I was not long in deciding that the heart *would not be accelerated* but a trifle—say a tenth—and, under the circumstances, I said: "The air *is* an anæsthetic."

From this rapid course of argument, I was so profoundly convinced of its truth, that without having first tried it upon my own person, I would have sat where I was, upon the curbstone, and had a tooth removed, with the perfect expectation of absence of pain and of still being conscious of touch. While yet walking, I commenced to breathe as rapidly as possible, and, as anticipated, found my steps growing shorter and shorter, until I came to a stand, showing to my mind clearly that my argument in advance was right, so far as locomotion was concerned; and, upon referring to my pulse, I found but little acceleration.

To what other conclusion could I arrive from this argument, with the foundation laid nineteen years before, when I established on my own person by experiment the fact of analgesia as induced from chloroform, with the many experiments in rapid respiration on tooth bone?

From this moment until its first application to the extraction of a tooth you can well imagine my suspense. That I might not fail in the very first attempt, I compelled myself and others in my household to breathe rapidly to investigate the phenomenon. This gave me some method of proceeding in its administration.

The first case soon appeared, and was a perfect success, going far beyond my anticipations, for the effect was such as to produce a partial paralysis of the hands and arms to the elbow. Again and again I tried it in every case of extraction and many other experiments, doubting my own senses for a long time, at a result so anomalous and paradoxical. I was reminded just here of a phenomenon which gave me additional proof—that of blowing a dull fire to revive it. For a minute or so one blows and blows in rapid succession, until, rising from the effort, a sense of giddiness for a few moments so overcomes that the upright position is with difficulty maintained. In this condition you are fitted for having a tooth extracted or an abscess lanced.

Believing that I had something new to offer which might be of use to suffering humanity, I read the first article upon it November 17, 1875, before the Franklin Institute. Shortly after I was invited before the Northern Medical Society of this city to address them thereon. A number of medical gentlemen have been using it in their practice, while the bulk of them have spurned it as "negative" and preposterous, without any effort at trying it, which I can *now* very well understand.

Unless one is aware of the fact that in the use of any agent which has the power to suspend the volition, it can be taken to that point where he is still conscious of *touch and hearing*, and at the same time not cognizant of pain inflicted, the action of rapid breathing can not be understood. And I regret to say that of three-fourths of the medical men I have talked with on the subject they had not been aware of such a possibility from ether and chloroform. Until this analgesic state could be established in their minds it was impossible to convince them that the exeess of oxygen, as obtained by rapid breathing, could be made to produce a similar effect. I should have been as reluctant as anyone to believe it, had I not personally experienced the effect while performing an operation which would otherwise have been very painful. Such a result could not well be reached by any course of reasoning alone.

Has it proven in my practice what has been claimed for it—a substitute for the powerful anæsthetics in minor operations in surgery? Most emphatically, yes! So completely has it fulfilled its humble mission in my office, that I can safely assert there has not been more than five per cent. of failures. I have given it under all circumstances of diseased organs, and have seen no other than the happiest results in its after effects. It may well be asked just here: Why has it not been more generally and widely nsed by the dental profession as well as the medical, if it is really what is claimed for it? The most satisfactory and charitable answer to be given is, the failure upon their part to comprehend the *fact*, as existing in chloroform and ether, that there is such a state as analgesia; or, in other words, that the animal economy is so organized, while the sense of touch is not destroyed, but rather increased, the mind of the subject fails to perceive a sense of pain when anæsthetics are given, and the effects are manifested in the primary stage. As I before intimated, such is the knowledge possessed by most of those who administer ether and chloroform. This was enough to cause nearly every one to look upon it as a bubble or air castle. Many gentlemen told me they tried it upon themselves, and, while it affected them very seriously by giddiness, they still *retain consciousness*; and, such being the case, no effect could be produced for obtunding pain. Others told me they were afraid to continue the breathing, alarmed at the vertigo induced.

And the practitioner who has adopted it moie effectively than any other laughed at me when I first told him of the discovery; but his intimate association with me changed his views after much explanation and argument between us.

It was hardly to be expected that without any explanation from me as to the *modus operandi* of rapid breathing, other than a few suggestions or directions as to how the effect was induced, even the most liberal of medical men should be able to make it effective, or have the least disposition to give it a preliminary trial upon themselves, and, of course, would not attempt it upon a patient. Notwithstanding, it found a few adherents, but only among my personal medical friends, with whom I had an opportunity to explain what I believed its physiological action, and the cases of success in my own practice. To this I have submitted as among the inevitable in the calendar of discoveries of all grades.

My own profession have attempted to *ridicule* it out of its birthright and possible existence, which style of argument is not resorted to by true logicians.

To all this I can truly say I have not for one moment faltered. I could afford to wait. The liberality of this society alone fully compensates for the seeming indisposition of the past, believing that it is proper that every advance should be confronted, and, if in time found worthy, give it God speed.

From its first conception I have diligently labored to solve its *modus operandi*, and the doubt in my own mind as to whether I could be mistaken in my observations. I asked the opinion of our best chemical teachers if air could have such effect. One attributed it to oxygen stimulation, and the other to nitrogen. Another gentleman told me the medical profession had come to the conclusion, that it was possible for me to thus extract teeth, but it was due solely to my strong *personal magnetism*, (which power I was not aware I possessed.)

Now, from what I have related of the successive and natural steps which finally culminated in this process or plan of analgesia induced by an excess of ordinary air taken forcibly into the lungs above what is necessary for life, and from what I shall state as to the apparent anomalous or paradoxical effects, with its physiological action, and the simple tests made upon each of my patients, I shall trust to so convince you of its plausibility and possibility that it will be made use of in hundreds of minor operations where ether and chloroform are now used.

Aside from my assertion and that of its friends, that the effects can be produced by air alone, you must have some light shed upon the causes of its physiological action, which will appeal to your *medical* reason.

To assign an action to any drug is difficult, and in the cases of ether and the other anæsthetics a quarter of a century still finds many conflicting opinions. This being true, you will deal leniently with me for the opinion I hold as to their analgesic action. Of course it will be objected to, for the unseen is, to a great extent, unknowable. Enough for my argument, however; it seems to suit the case very well without looking for another; and while it was based on the phenomenon resulting from many trials, and not the trials upon it as a previous theory, I shall be content with it until a better one can be found.

What is it I claim as a new discovery, and the fact and its philosophy.

I have asserted that I can produce, from rapidly breathing common air at the rate of a hundred respirations a minute, a similar effect to that from ether, chloroform, and nitrous oxide gas, in their primary stages; and I can in this way render patients sufficiently insensible to acute pain from any operation where the time consumed is not over twenty to thirty seconds. While the special sense of pain is obtunded, and in many cases completely annulled, consciousness and general sensibility being preserved.

To accomplish this, each patient must be instructed how to act and what to expect. As simple as it may seem there is a proper and consistent plan to enable you to reach full success. Before the patient commences to inhale he is informed of the fact that, while he will be unconscious of pain he will know full or partially well every touch upon the person; that the inhalation must be vigorously kept up during the whole operation without for an instant stopping; that the more energetically and steadily he breathes, the more perfect the effect, and that if he cease breathing during the operatian, pain will be felt. Fully impress them with this idea, for the very good reason that they may stop when in the midst of an operation, and the fullest effects be lost. It is obligatory to do so on account of its evanescent effects, which demand that the patient be pushed by the operator's own energetic appeals to "go on." It is very difficult for any person to respire more than one hundred times to the minute, as he will become by that time so exhausted as not to be able to breathe at all, as is evidenced by all who have thus far followed my directions. For the next minute following the completion of the operation the subject will not breathe more than once or twice. Very few have force enough left to raise hand or foot. The voluntary muscles have nearly all been subjugated and overcome by the undue effort at forced inhalation of one hundred instead of seventeen, the normal standard. It will be more fully understood further on in my argument why I force patients, and am constantly speaking to them to go on.

I further claim that for the last four years, so satisfactory has been the result of this system in the extracting of teeth and deadening extremely sensitive dentine, there was no longer any necessity for chloroform, ether, or nitrous oxide in the dental office. That such teeth as cannot be extracted by its aid can well be preserved and made useful, except in a very few cases, who will not be forced to breathe.

The anæsthetics, when used in major operations, where time is needed for the operation, can be made more effective by a lesser quantity when given in conjunction with

"rapid breathing." Drs. Garrettson and Hewson, who have thus tried it, tell me it takes one-half to three-fourths less, and the after effects are far less nauseating and unpleasant.

As an agent in labor, where an anæsthetic is indicated, it is claimed by one who has employed it (Dr. Hewson) in nearly every case for three years, he has used 'rapid breathing" solely, and to the exclusion of chloroform and ether. For this I have his assertion, and have no doubt of it whatever, for if any agent could break down the action of the voluntary muscles of the parts involved, which prevent the involuntary muscles of the uterus from having their fullest effect, it is this. The very act of rapid breathing so affects the muscles of the abdomen as to force the contents of the uterus downward or outward, while the specific effect of the air at the end of a minute's breathing leaves the subject in a semi prostrate condition, giving the uterus full chance to act in the interim, because free of the will to make any attempt at withholding the involuntary muscles of the uterus from doing their natural work. It is self-evident; and in this agent we claim here a boon of inestimable value. And not last in such cases is, there is no danger of hemorrhage, since the cause of the effect is soon removed.

In attestation of many cases where it has been tried, I have asked the mother, and, in some cases, the attendants, whether anything else had been given, and whether the time was very materially lessened; there has been but one response, and that in its favor.

Gentlemen, if we are not mistaken in this, you will agree with me in saying that it is no mean thing, and should be investigated by intelligent men and reported upon. From my own knowledge of its effects in my practice, I am bound to believe this gentleman's record.

I further claim for it a special application in dislocations. It has certain peculiar merits here, as the will is so nearly subjected by it as to render the patient quite powerless to resist your effort at replacing, and at the same time the pain is subdued.

It is not necessary I should further continue special applications; when its *modus operandi* is understood, its adaptation to many contingencies will of a sequence follow.

It is well just here, before passing to the next point of consideration, to answer a query which may arise at this juncture:

What are the successive stages of effects upon the economy from its commencement until the full effect is observed, and what proof have I that it was due to the amount of air inhaled?

The heart's action is not increased more than from seventy (the average) to eighty and sometimes ninety, but is much enfeebled, or throwing a lesser quantity of blood. The face becomes suffused, as in blowing a fire or in stooping, which continues until the breathing is suspended, when the face becomes paler. (Have not noticed any purple as from asphyxia by a deprivation of oxygen.) The vision becomes darkened, and a giddiness soon appears. The voluntary muscles furthest from the heart seem first to be affected, and the feet and hands, particularly the latter, have a numbness at their extremities, which increases, until in many cases there is partial paralysis as far as the elbow, while the limbs become fixed. The hands are so thoroughly affected that when open, the patient is powerless to close them and *vice versa.* There is a vacant gaze from the eyes and looking into space without blinking of the eyelids for a minute or more. The head seems incapable of being held erect, and there is no movement of the arms or legs as is usual when in great pain. There is no disposition on the part of the patient to take hold of the operator's hand or interfere with the operation.

Many go on breathing mechanically after the tooth has been removed, as if nothing had occurred. Some are aware that the tooth has been extracted, and say they felt it; others could not tell what had been accomplished.

The majority of cases have an idea of what is being done, but are powerless to resist.

With the very intelligent, or those who stop to reason, I have to teach them the peculiarities of being sensible of touch and not of pain.

[TO BE CONTINUED.]

